# Supplementary material for: Study of correlations between serum taurine, thyroid hormones and echocardiographic parameters of systolic function in clinically healthy Golden retrievers fed with commercial diet
Source: PLoS One. 2024 May 16;19(5):e0297811. doi: 10.1371/journal.pone.0297811 (PMC11098416; doi:10.1371/journal.pone.0297811)
Supplement: S1 Table — (DOCX) [file pone.0297811.s001.docx]

**Supporting information**

**S1 Table**

|  |  | **Mean** | **SD** | **Normal range** |
| --- | --- | --- | --- | --- |
| **CBC** | RBC (10^6/µL) | 6.00 | 0.67 | 5.7-8.8 |
|  | HCT (%) | 42.40 | 3.69 | 37.1-57 |
|  | HGB (g/dL) | 14.82 | 1.40 | 12.9-18.4 |
|  | MCHC (g/dL) | 34.93 | 0.85 | 19.5-24.2 |
|  | %RETICOL | 0.75 | 0.35 | <1 |
|  | WBC (K/µL) | 12.82 | 1.24 | 6-13.9 |
|  | NEU (K/µL) | 8.10 | 3.23 | 3-11.5 |
|  | EOS (K/µL) | 0.46 | 0.31 | 0.1-1.2 |
|  | LYMPH (K/µL) | 3.43 | 1.10 | 1-4.8 |
|  | MONO (K/µL) | 0.81 | 0.45 | 0.1-1.5 |
|  | BAS (K/µL) | 0.04 | 0.06 | Rare |
|  | PLT (K/µL) | 173.67 | 75.40 | 143-400 |
| **Biochemical analysis** | CREA (mg/dl) | 1.21 | 0.21 | <1.5 |
|  | ALP (U/L) | 64.00 | 28.25 | <180 |
|  | ALB (g/dl) | 3.11 | 0.27 | 2.3-3.5 |
|  | UREA (mg/dl) | 34.90 | 11.03 | 20-60 |
|  | ALT (U/L) | 42.00 | 22.91 | <60 |
|  | TP (g/dl) | 6.39 | 0.61 | 5.4-7.5 |

RBC: red blood cells; HCT: hematocrit; HGB: hemoglobin, MCHC: mean corpuscular hemoglobin Concentration; RETICOL: reticulocytes; WBC: white blood cells; NEU: neutrophils; EOS: eosinophils; LYMPH: lymphocytes; MONO: monocytes; BAS: basophils; PLT: platelets; CREA: creatinine; ALP: alkaline phosphatase; ALB: albumins; UREA: urea; ALT: alanine aminotransferases; TP: total proteins.
