# Supplementary material for: Study of correlations between serum taurine, thyroid hormones and echocardiographic parameters of systolic function in clinically healthy Golden retrievers fed with commercial diet
Source: PLoS One. 2024 May 16;19(5):e0297811. doi: 10.1371/journal.pone.0297811 (PMC11098416; doi:10.1371/journal.pone.0297811)
Supplement: S2 Table — (DOCX) [file pone.0297811.s002.docx]

**S2 Table**

| **Animal ID** | **Number of complete generations** | **Number of incomplete generations** | **Inbreeding** |
| --- | --- | --- | --- |
| 1 | 6 | 15 | 0.298150033 |
| 2 | 6 | 11 | 0.28968811 |
| 3 | 5 | 12 | 0.107135773 |
| 4 | 5 | 9 | 0.064178467 |
| 5 | 5 | 14 | 0.052016497 |
| 6 | 6 | 11 | 0.046703339 |
| 7 | 6 | 11 | 0.046703339 |
| 8 | 6 | 11 | 0.046703339 |
| 9 | 6 | 11 | 0.046703339 |
| 10 | 6 | 11 | 0.046703339 |
| 11 | 5 | 13 | 0.045406818 |
| 12 | 6 | 13 | 0.042559147 |
| 13 | 6 | 13 | 0.037097216 |
| 14 | 6 | 14 | 0.034627527 |
| 15 | 6 | 14 | 0.034403265 |
| 16 | 6 | 13 | 0.033360124 |
| 17 | 7 | 13 | 0.033240318 |
| 18 | 5 | 12 | 0.022294044 |
| 19 | 7 | 15 | 0.017852619 |
| 20 | 7 | 15 | 0.017852619 |
| 21 | 5 | 11 | 0.01782608 |
| 22 | 5 | 12 | 0.017715454 |
| 23 | 5 | 12 | 0.017562866 |
| 24 | 5 | 12 | 0.015255928 |
| 25 | 5 | 15 | 0.015235193 |
| 26 | 4 | 12 | 0.01470089 |
| 27 | 6 | 16 | 0.013279572 |
| 28 | 4 | 15 | 0.012434006 |
| 29 | 4 | 11 | 0.012107849 |
| 30 | 6 | 12 | 0.011738062 |
| 31 | 5 | 13 | 0.010973334 |
| 32 | 6 | 14 | 0.009297848 |
| 33 | 5 | 11 | 0.008388519 |
| 34 | 5 | 13 | 0.007175565 |
| 35 | 5 | 13 | 0.007175565 |
| 36 | 5 | 13 | 0.007175565 |
| 37 | 6 | 13 | 0.005191147 |
| 38 | 5 | 12 | 0.00378418 |
| 39 | 4 | 15 | 0.003641725 |
| 40 | 4 | 15 | 0.003641725 |
| 41 | 6 | 11 | 0.003368378 |
| 42 | 3 | 11 | 0.001960754 |
| 43 | 4 | 15 | 0.001449585 |
| 44 | 5 | 16 | 0.001265466 |
| 45 | 4 | 14 | 0.001255989 |
| 46 | 4 | 12 | 0.00038147 |
| 47 | 6 | 13 | 0.000272751 |
| 48 | 4 | 11 | 0.000259399 |
| 49 | 4 | 15 | 0 |
| 50 | 4 | 16 | 0 |
